# The Biosafety and Risk Management in Preparation and Processing of Cerebrospinal Fluid and Other Neurological Specimens With Potential Coronavirus Infection

**DOI:** 10.3389/fneur.2020.613552

**Published:** 2021-01-20

**Authors:** Chien-Chin Chen, Pei-Chun Chiang, Tsung-Hsien Chen

**Affiliations:** ^1^Department of Pathology, Ditmanson Medical Foundation Chia-Yi Christian Hospital, Chiayi, Taiwan; ^2^Department of Cosmetic Science, Chia Nan University of Pharmacy and Science, Tainan, Taiwan; ^3^Department of Internal Medicine, Ditmanson Medical Foundation Chia-Yi Christian Hospital, Chiayi, Taiwan

**Keywords:** COVID-19, CSF, neurology, coronavirus, cytology, biosafety, myopathy, neuromuscular

## Abstract

The recent outbreak of coronavirus disease 2019 (COVID-19), caused by Severe Acute Respiratory Syndrome Coronavirus 2, has become a global threat. Due to neurological manifestations presented throughout the coronavirus disease process, the potential involvement of COVID-19 in central nervous system has attracted considerable attention. Notably, the neurologic system could be widely affected, with various complications such as acute cerebrovascular events, encephalitis, Guillain-Barré syndrome, and acute necrotizing hemorrhagic encephalopathy. However, the risk assessment of exposure to potential biohazards in the context of the COVID-19 pandemic has not been clearly clarified regarding the sampling, preparation, and processing neurological specimens. Further risk managements and implantations are seldom discussed either. This article aims to provide current recommendations and evidence-based reviews on biosafety issues of preparation and processing of cerebrospinal fluid and neurological specimens with potential coronavirus infection from the bedside to the laboratory.

## Introduction

The Severe Acute Respiratory Syndrome Coronavirus-2 (SARS-CoV-2) has caused the coronavirus disease 2019 (COVID-19) pandemic, an illness with the high transmissibility and a broad spectrum of clinical manifestation. As of December 15, 2020, the World Health Organization (WHO) reported more than 70 million cases and over 1.6 million deaths globally in the COVID-19 pandemic (1). COVID-19 is the third epidemic of human coronavirus diseases after the severe acute respiratory syndrome (SARS) in November 2002, and the Middle East respiratory syndrome (MERS) in September 2012 (2). In comparison with other epidemic coronaviruses, SARS-CoV-2 is less lethal but far more transmissible than MERS coronavirus (MERS-CoV) and SARS coronavirus (SAR-CoV) (3, 4). It is believed that SARS–CoV-2 can spread by respiratory droplets, unprotected direct contact with patients, and touching contaminated objects (5, 6). Since symptoms of COVID-19 can be in a wide variety of severity, medical professionals are in particular at risk of exposure to SARS-CoV-2 through close contact via respiratory droplets and contaminated surface and direct handling of contagious materials from patients with COVID-19 (7). With regard to the safe collecting and handling clinical specimens in the pandemic, a few reports have emphasized the need for the worldwide standardization of biosafety protocols (5, 8, 9). Notably, the neurological manifestation and morbidities of COVID-19 have been widely reported (10–16). Mao et al. (15) reported that neurologic symptoms were present in 36.4% of all patients with COVID-19, especially more frequent in patients with severe illness. Moreover, in a patient with acute cerebellitis, the viral RNA of SARS-CoV-2 was detected in his oropharynx, nasopharynx, and cerebrospinal fluid (CSF) (17). However, the biosafety and risk assessment in preparation and processing of CSF and other neurological specimens were seldom discussed. This mini-review aims to provide an integrative, evidence-based review to guide the preparation and processing of neurological specimens with potential coronavirus infection and therefore to prevent nosocomial infection.

## Coronaviruses and Neurologic Diseases

Although COVID-19 primarily presents as a respiratory disease, SARS-CoV-2 affects multiple organs or systems, including the central nervous system (CNS), peripheral nervous system (PNS), and neuromuscular system (15, 18–20). In a large case cohort of COVID-19, 24.8% had CNS symptoms (e.g., dizziness, headache, and impaired consciousness), 8.9% had PNS symptoms, and 10.7% had skeletal muscle injury (15). In a nationwide surveillance of 125 patients with COVID-19 and neurological or psychiatric disease, 62% of them had a cerebrovascular event, while 31% of them presented with altered mental status (19). Similarly, the epidemic of SARS was reported with various neurological complications including encephalopathy, seizures, stroke, cranial nerve palsies, peripheral neuropathy, and myopathy (20–24). Also, patients with MERS were occasionally presented to have neurological symptoms and neuromuscular complications (24–27). The prevalence of CNS complications reached 0.04% for SARS and 0.20% for MERS, and besides the prevalence of PNS complications was 0.05% for SARS and 0.16% for MERS (14).

Although the mechanism of CNS involvement of COVID-19 remains unclear, there is a three-step model which refers to viral neuroinvasion, CNS clearance, and immune response (28). In the first stage, SARS-CoV-2 may enter the brain via the bloodstream and/or transcribriform route along the olfactory nerve, and the viral load in CSF should increase (28). The respiratory symptoms are minimal in the early stage. With the interaction between the spike protein S1 of SARS-CoV-2 and the host angiotensin-converting enzyme 2 receptor (ACE2), SARS-CoV-2 may enter both glial and neuronal cells (29). In some cases, the neuroinvasion may cause a direct neuronal damage and subsequently result in neurological symptoms. Moreover, the consumption and downregulation of ACE2 by SARS-CoV-2 virus may lead to imbalance of the renin angiotensin system resulting in endothelial dysfunction, vasoconstriction, and subsequently ischemic events (30). In the second stage, SARS-CoV-2 may infect the brainstem affecting the respiratory drive. The viral load in respiratory secretions would increase predominantly, but the viral load in CSF significantly decreases. The CSF clearance of SARS-CoV-2 may greatly contribute to a low virus detection rate in CSF samples from patients with COVID-19 and CNS involvement. In the third stage, an immuno-mediated CNS damage may form, since SARS-CoV-2 can trigger the production of antibodies against glial cells, as a para-infective or post-infective phenomenon (28). In consequence, the respiratory system would be severely affected and cause neurotoxic hypoxia with subsequent brain damage (28).

With regard to neuromuscular involvement of SARS-CoV-2, myositis, acute myelitis, Guillain Barre syndrome, Miller Fisher syndrome, polyneuritis cranialis, oculomotor paralysis and Bell's Palsy have been discussed to be associated with COVID-19 (18, 30–34). On electrodiagnostic testing, most of the abovementioned patients had demyelinating pattern, some had acute sensory motor axonal neuropathy, and few had acute motor axonal neuropathy (18). In a patient with COVID-19 and myositis, the muscle biopsy revealed inflammatory infiltration around vessels and endomysial extension, regeneration of muscular fibers, and elevated HLA Class ABC expression (33). The exact mechanism remains unknown, although a few hypothetic theories were proposed, including ACE2 mediated pathway, olfactory pathway, trans-synaptic pathway, and immune mediated pathway (18). Since the muscle cells express ACE2, the direct invasion by the SARS-CoV-2 entering the muscle cells via the ACE2 may be possible (30). In addition, cytokine storms in the advanced phase of COVID-19 could lead to immune-mediated muscle damages (30).

## The Clinical Sampling and Preparation: Lumbar Puncture and Muscle Biopsy

Lumbar puncture (LP) is a medical procedure at the level of L2 to L5 vertebrae to collect CSF for examining infectious, inflammatory, and neoplastic diseases involving the CNS. In viral encephalitis, there is usually a mild to moderate CSF pleocytosis with predominant lymphocytes, normal glucose ratio, and slightly elevated protein (14, 35). The standard of diagnosing a CNS viral infection is to demonstrate the virus in the CNS, either from culture or polymerase chain reaction (PCR) of brain tissue or CSF.

Muscle biopsy (MB) is important for the evaluation and diagnosis of patients who are suspected of having an underlying neuromuscular disorder (36). With an open biopsy or needle biopsy technique under local anesthesia, bundles of skeletal muscles are taken for the required tests, including frozen sections for enzyme histochemistry, paraffin embedding for muscle fiber morphology and inflammatory patterns, electron microscopy for ultrastructural analysis, and biochemical testing for assessing storage and mitochondrial diseases (36).

In the pandemic, to perform a LP or a MB might be at risk to expose coronaviruses, since direct contact or respiratory droplets might be infectious. Since both LP and MB are time-consuming, the performer and all teammates would expose to patients' droplet aerosols in a poorly ventilated room. In closed rooms, the SARS-CoV-2 can be detectable in aerosols for 3 h and persists on surfaces (such as cardboard, stainless steel, and plastic surfaces) from 24 to 72 h (6). Thus, the sampling or collecting biological materials from patients should be careful and need to follow the recommendations or guidelines in the pandemic (5, 37–39). First, a site-specific and activity-specific risk assessment should be regularly performed to ensure the competency level of the healthcare workers, the equipment and facility, and the resources that are available. Meanwhile, clinical triage should be ensured by assessing all patients for early detection of COVID-19, and immediate isolation of patients with suspected COVID-19 in an area separate from others (37). Regarding the environment, LP and MB should be performed in an adequately ventilated room with at least of 60 liters/s/patient air flow (37). The environmental cleaning and disinfection procedures should be consistently and correctly performed. Notably, coronaviruses can be inactivated by surface disinfectants with 62–71% ethanol (C_2_H_6_O), 0.5% hydrogen peroxide (H_2_O_2_) or 0.1% sodium hypochlorite (NaClO) within 1 min (40).

Although LP and MB are not aerosol-generating procedures, the neurological professionals should wear a medical mask, eye protection (goggles) or facial protection (face shield), a clean long-sleeved gown, and gloves (37). After procedures, personal protective equipment and wastes should be properly disposed, and hand hygiene should be performed before and after contact with each patient. Lastly, it is important to clean and disinfect the surfaces that the patient was in contact with. With regard to the transportation, CSF or muscle for virus detection can be shipped at 2–8°C and delivered promptly to the laboratory (41). Notably, patient specimens from suspected or confirmed SARS-CoV-2 infection should be transported as UN3373, “Biological Substance Category B” (42, 43). All the biosafety recommendations are summarized in Table 1.

**Table 1:** A summary of biosafety recommendations to prevent coronavirus (COVID-19) for lumbar puncture and muscle biopsy.

Before and after lumbar puncture or muscle biopsy	1. Ensure clinical triage and assess patients for early recognition of COVID-19 infection. 2. Conduct a site-specific and activity-specific risk assessment with appropriate risk control measures in place. 3. Have adequate PPE supplies in sufficient quantity. 4. Perform procedures in an adequately ventilated room with at least of 60 L/s/patient air flow. 5. Wear standard medical masks, eye protection (goggles) or facial protection (face shield), long-sleeved gowns, and gloves. 6. The process of contact with each patient, properly dispose of all PPEs and wastes and perform hand hygiene. 7. Clean and disinfect the surfaces that the patient was in contact with.
Precautions of transportation	1. Patient specimens from suspected or confirmed SARS-CoV-2 infection should be transported as UN3373, “Biological Substance Category B”. 2. Deliver all specimens promptly by hand whenever possible. 3. Ensure that all personnel who transport specimens have received training in safe handling practices and spill decontamination procedures. 4. Timely notify the laboratory and correctly label the specimen with informative request forms.
Process samples	1. Diagnostic tests, such as cytology, biochemistry, and formalin-fixed paraffin sections, should be handled in a BSL-2 laboratory. 2. Wear and remove PPE properly, as determined by a detailed risk assessment, with hand hygiene. 3. Processing of all specimens before inactivation should take place in a validated biological safety cabinet or primary containment device. 4. All procedures should be performed in a manner that minimizes the generation of aerosols and droplets. 5. During processing, appropriate disinfectants with proven activity against coronaviruses should be used.

*COVID-19, coronavirus disease 2019; SARS-CoV-2, severe acute respiratory syndrome coronavirus-2; PPE, personal protective equipment; BSL-2, biosafety level 2*.

## To Process CSF and Other Neurological Specimens

Since all specimens collected for laboratory investigations should be considered potentially infectious, all procedures must be performed according to risk assessment and strategies for biosafety (42, 43). Before inactivation of all specimens, the initial processing should be performed in a validated biological safety cabinet or primary containment device (42). In addition to detecting viruses by sequencing or PCR, all diagnostic laboratory works for neurological specimens, including biochemistry, cytology, and special stains should be performedat a facility using procedures equivalent to Biosafety Level 2 (BSL-2) (42, 43). In the light of inactivation of coronaviruses, fixatives with ethanol concentrations of 78%-95% for at least 30 s could inactivate SARS-CoV, and either 10% formalin or 4% paraformaldehyde for at least 30 min would efficiently inactivate MERS-CoV–infected cells (9). Alcohol fixed preparation also lyses red blood cells, reducing the risk of viremia. The abovementioned fixations are the reasons why specimens with Papanicolaou staining or formalin fixation can be taken as inactivated (9). Moreover, the external lysis buffer of common RNA extraction kits for viral detection is effective to inactivate SARS-CoV-2 without heat or other additional methods (42).

Currently, the identification of viral RNA through nucleic acid amplification technologies, such as reverse-transcription polymerase chain reaction (RT-PCR) in a patient's biological samples, remains the gold standard for identifying infections with coronaviruses. Notably, SARS-CoV was detected in CSF by RT-PCR in two cases of encephalopathy (44, 45) and was cultured from brain tissues of an autopsy case (46). In the COVID-19 pandemic, although the neurological manifestations were not uncommon, SARS-CoV-2 RNA was rarely detected in CSF by RT-PCR (Table 2) (17, 47–65). And, to the best of our knowledge, there is no MB specimen demonstrating the evidence of SARS-CoV-2 infection via culture or RT-PCR. Based on the relative frequencies of detectable SARS-CoV-2 RNA in different samples from published reports, Chen and Chi (5) suggested to categorize the cytological and pathological samples into the high risk, intermediate risk, and low risk groups. Accordingly, CSF and MB specimens can be categorized into the low risk group with limited evidence of SARS-CoV-2 RNA detection and should subsequently follow the principles of good microbiological practices and procedures to be handled (5). Although the presence of viral RNA is not equivalent to live infectious viruses, RT-PCR is an important method to identify infectious agents (66).

**Table 2:** The detection of SARS-CoV-2 in CSF from patients with COVID-19 and neurological symptoms.

**References**	**Age/Gender**	**Neurological symptoms and/or diagnoses**	**No. of CSF positive/total patients**	**Note**
Fadakar et al. (17)	47/Male	Progressive vertigo, headache, and ataxia. Acute cerebellitis.	1/1	SARS-CoV-2 RNA was detected in oropharyngeal, nasopharyngeal, and CSF specimens. The CSF analysis demonstrated mild pleocytosis (80% lymphocyte), normal glucose (60 mg/dL), elevated protein (58 mg/dL) and lactate dehydrogenase (134 u/L), and negative results in Gram stain, culture, and cytology.
Moriguchi et al. (47)	24/Male	Fever, unconsciousness, and neck stiffness. Meningitis/encephalitis.	1/1	SARS-CoV-2 RNA was not detected in the nasopharyngeal swab, but shown in CSF. The CSF cell count showed pleocytosis.
Cebrián et al. (48)	74/Female	Severe headache with photophobia, vomiting, and progressive confusion.	1/1	Both nasopharyngeal and CSF tests for SARS-CoV-2 RNA were positive. The CSF analysis yielded no specific finding (1 leukocyte/mm^3^, 1 red blood cell/mm^3^, protein: 30 mg/dL, glucose: 82 mg/dL, and opening pressure: 10 cmH_2_O). Acid-fast bacilli and bacterial cultures and stains were negative.
Domingues et al. (49)	42/Female	Paresthesias of the left upper limb, left hemithorax, and hemiface. Demyelinating disease.	1/1	SARS-COV-2 RNA was positive in the first CSF sample, but negative in nasal and pharyngeal samples. The CSF analysis showed 1 leukocyte/mm^3^, protein of 32 mg/dL, and glucose of 68 mg/dL.
El-Zein et al. (50)	40/Male	Visual hallucinations, forgetful, and confusion with orientation. Meningoencephalitis.	0/1	SARS-CoV-2 was positive in a nasopharyngeal swab, but negative in CSF. The CSF studies showed lymphocytic pleocytosis, elevated glucose (70 mg/dL), and decreased protein levels (19 mg/dL).
Lu et al. (51)	51/Male	Fever, pharyngalgia, excited, talkative, irritable, and energetic. COVID-19 with manic-like symptoms.	0/1	SARS-CoV-2 RNA was positive in sputum and stool, but negative in CSF. SARS-CoV-2 specific IgG antibody in CSF was positive. The CSF analysis demonstrated no pleocytosis and normal protein.
Vandervorst et al. (52)	29/Male	General weakness, dry cough, dyspnea, confusion, and disorientation. Encephalitis.	0/1	SARS-CoV-2 was positive in a nasopharyngeal swab, but negative in CSF. The CSF cell count, protein and glucose levels were within normal limits. Anti-SARS-CoV-2 IgM and IgG's were negative in CSF.
Sun and Guan (53)	N/A	N/A	1/1	CSF was tested positive for SARS-CoV-2 by gene sequencing.
Al Saiegh et al. (54)	31/Male and 62/Female	Male: severe headache and loss of consciousness with aneurysmal subarachnoid hemorrhage. Female: right hemiparesis and aphasia with a left middle cerebral artery occlusion.	0/2	Both patients' nasal swabs were positive for SARS-CoV-2 RNA, but both patients' CSF specimens were negative. No other data of CSF analysis were provided.
Bodro et al. (55)	25/Male and 49/Male	25-year-old: headache, left-sided paresthesias, and ipsilateral paresis. 49-year-old: fever, myalgia, temporospatial disorientation, confusion, and agitation.	0/2	SARS-CoV-2 RNA was positive in the nasopharyngeal swabs of both cases, but negative in CSF of both cases. In both patients, the CSF showed lymphocytic pleocytosis and increased proteins.
Benameur et al. (56)	31/Female, 34/Male, and 64/Male	31-year-old: respiratory failure, encephalitis and myelitis. 34-year-old: respiratory failure, encephalopathy with myoclonus. 64-year-old: respiratory failure and encephalopathy.	0/3	SARS-CoV-2 RNA was positive in the nasopharyngeal swabs of all 3 cases, but negative in CSF of all cases. The CSF data showed mild to markedly increased IgM for SARS-CoV-2 S1 protein in all 3 cases. And only one of three had neutrophilic pleocytosis with a high protein level.
Delorme et al. (57)	72/Male, 66/Female, 60/Female, and 69/Male	New-onset cognitive disturbances with central focal neurological signs or seizures.	0/4	SARS-CoV-2 RNA was positive in the nasopharyngeal swabs of all 4 cases, but negative in CSF of all cases. None of them had MRI features of encephalitis or significant CSF abnormalities.
Neumann et al. (58)	18 males and 12 females with a median age of 65.5 years	Altered mental state (33.3%), new paresis (30.0%), impaired consciousness (23.3%), hypo-/areflexia (30.0%), anosmia/hyposmia or ageusia/hypogeusia (20.0%), and seizures (16.7%).	0/30	In all 30 cases, RT-PCR tests for SARS-CoV-2 RNA from CSF were negative, although positive in all orophyryngeal swabs. Their CSF showed normal or slightly increased white blood cell count ( ≤ 8/μl) in 28 cases, while the CSF blood albumin ratio was normal in most cases.
Guilmot et al. (59)	12 males and 3 females with a median age of 62 years	Cranial neuropathy, coma, neuropsychiatric symptoms, delirium, and acute cerebrovascular disease.	0/13 (not perform in 2 patients with anticoagulation)	PCR for SARS-CoV-2 on the CSF was negative in all patients. The CSF lymphocytic pleocytosis was present only in two cases, one with anti-Caspr2-associated limbic encephalitis and the other with para-infectious polyradiculitis.
Uncini et al. (60)	27 males and 15 females with a median age of 57.5 years	Guillain- Barré syndrome, presenting hypoareflexia (80.9%) and limb weakness (76.2%).	0/25	In 42 cases with Guillain- Barré syndrome and SARS- CoV-2 infection, the SARS-CoV-2 RNA in CSF was negative in all 25 cases in whom was done. The CSF albuminocytological dissociation was found in 28/36 (77.8%).
Abu-Rumeileh et al. (61)	50 males and 23 females with a mean age of 55 years	Guillain- Barré syndrome with fever (73.6%), cough (72.2%), dyspnea (63.8%), hypo-/ageusia (22.2%), hypo-/anosmia (20.8%), and diarrhea (18.1%).	0/31	Only 31 cases were tested for SARS-CoV-2 RNA in CSF which was undetectable in all tested patients. The CSF albuminocytological dissociation was present in 71.2% of the cases (42/59). Mild pleocytosis, with a maximum cell count of 13/μl, was evident in 8.5% of cases (5/59).
Espíndola et al. (62)	N/A	Meningoencephalitis (1), encephalitis (1), facial palsy (2), delirium (2), intracranial hypertension (1), new daily persistent headache (1).	0/8	The CSF data revealed normal or mild elevated protein levels, while pleocytosis was particularly observed in the cases of meningoencephalitis.
Miller et al. (63)	21 males and 6 females with an average age of 37.5 years	Fever (48%), altered mental status (22%), headache (15%), dyspnea (7%), anosmia (6%), and psychosis (4%).	0/8	Only 8 cases were tested for SARS-CoV-2 RNA in CSF which was undetectable in all tested patients. The CSF analyses revealed elevated white blood cell counts (12/27) and protein (14/27).
Edén et al. (64)	5 males and 1 female with a median age of 60 years	Encephalopathies (4/6), suspected meningitis (1/6), and dysgeusia (1/6).	3/6	Three of six cases tested for SARS-CoV-2 RNA in CSF showed low levels of positivity in the first time (Ct values 39.0, 38.0, and 37.2). But the second test for SARS-CoV-2 RNA was undetectable in all these CSF samples. None of the patients had CSF pleocytosis. The albumin ratio and IgG-index in CSF were within the normal range in all cases.
Khodamoradi et al. (65)	49/Female	Fever, headache, malaise, nausea, and vomiting. Meningitis.	1/1	The RT-PCR for COVID-19 was positive in CSF, but negative in oropharyngeal/nasopharyngeal samples. The CSF analysis showed pleocytosis, elevated protein levels, and normal glucose levels.

*CSF, cerebrospinal fluid; SARS-CoV-2, severe acute respiratory syndrome coronavirus-2; N/A, not available; MRI, magnetic resonance imaging; RT-PCR, reverse-transcriptase polymerase chain reaction*.

## Conclusion

With the growing number of confirmed COVID-19 cases, it is essential that the neurological experts and clinical laboratories implement clinical triage, drastic measures, and appropriate procedures and facilities for ensuring the safety and interests of valuable healthcare workers in times of the pandemic. The lessons learned from SARS and MERS could give us more insights to conduct efficient preventive measures in healthcare settings. Although LP and MB are important diagnostic procedures for CNS and neuromuscular diseases, neurological practitioners must be well-prepared and avoid of non-emergent procedures to prevent potential exposures to COVID-19. The collection, transportation, and processing of neurological specimens should warrant the use of WHO guidelines, academic recommendations, and BSL-2 procedures. Herein, although the biological safety and security issues were rarely discussed in neurology, we hope that both neurologists and laboratory professionals can benefit from this integrative mini-review in dealing with the COVID-19 crisis.

## Author Contributions

C-CC: study concept and design, acquisition of data, manuscript writing, critical revision of the manuscript for important intellectual content, and study supervision. P-CC: acquisition of data, analysis and interpretation, manuscript writing, and critical revision of the manuscript for important intellectual content. T-HC: critical revision of the manuscript for important intellectual content. All authors read and approved the final manuscript.

## Conflict of Interest

The authors declare that the research was conducted in the absence of any commercial or financial relationships that could be construed as a potential conflict of interest.
